# Supplementary material for: Chromothripsis during telomere crisis is independent of NHEJ, and consistent with a replicative origin
Source: Genome Res. 2019 May;29(5):737–49. doi: 10.1101/gr.240705.118 (PMC6499312; doi:10.1101/gr.240705.118)
Supplement: Supplemental Material [file supp_gr.240705.118_Supplemental_file_1.zip › contigs/annotated_contigs/DB90/contig.4.DB90_length_925_mean_cov_15.267027027.docx]

**DB90_length_925_mean_cov_15.267027027**

```
ATTTACGAAACAAACAGTATTAATAATACAGCAAATATCATACCTAATGGATTATTGAAATATTTTTTCTCTGAGGTTGGGAAGGAGAT
 >chr7:118885315-118885498 - E=4e-98
AAAGATGACTGCTACTACTTCCATTTAAAAAAACACAAGCAGCCTTTACTTGACCTAAAGGTAAAAATAAATTTAAGAAAACATGCAAA

TACAT|GTAC|ACTTAAGGTCGGCTTGCCCTTTCTCTTCACAAAAGAACTCTTCTAAGAGAGAGGAAAAATGCCTTCTCCCAGTCATCT
          >chr7:118882871-118883004 + E=4e-68
TCTGACCTTCTAAAGGCTTCGTCTTGGACTAACATAACTGAACAGGCCTTCC|AAG|GAGTGATTTTAACAATTCCATCCACCCATCTC
                                                     >chr7:118883163-118883432 + E=7e-1
CAACCCAATATCCCACAGTAGGGTGTTTTTACTGAGCTGACATCCTTGGAATGCATAGGTGCTAAGGCAAGAGTTTTCCTCAACATTAG
50
TTGATTATGGCCCTATATTTCCTTTGTTTTCCTATATAATACTTACAGAACTGTAAGGTATATGAAAAATTCCATGTTTAATTGCATAC

AACTCAGTTGATTTTCAGAAATGGAATGCACTCATGTAACTAGTAACTGGATC|AAG|TACACGAACTCCTATTAGACATGTGAAGGGA
                                                      >chr7:118882537-118882875 - E=2e-
TTGCTGTTGTAAAGATACTGTATAAAATATATAATAATAAGTACAGAAGGAAAAAATAGGAGAATTCTGTGAGTTTATGTTTTTTTATA
185
TGTTCTCCCAATTTCTTACGTTCTCATACACTGGATATTTTAGAACCCCAGACTCACTGTAAGAAACCCAACATTATCTCCAAAATACC

AAAACACACACACACACACACACACACACACACACTTTTTACCTCTTAGCCTTCATTTGTTTTGATGCCTTTATATTACTGTACTCTAA

AAAGGTCTAATTTATACATCCTAAGGTGAAACATGTACAAT
```
